# Association among early life stress, mood features, hopelessness and suicidal risk in bipolar disorder: The potential contribution of insomnia symptoms

**DOI:** 10.1192/j.eurpsy.2023.607

**Published:** 2023-07-19

**Authors:** L. Palagini, A. Gemignani, D. Riemann

**Affiliations:** ^1^University of Ferrara, Ferrara; ^2^Università di pisa, pisa, Italy; ^3^Univrsity of Freiburg, Freiburg, Germany

## Abstract

**Introduction:**

Mood disorders are complex illnesses possibly resulting from the interaction of genetic, physiological, psychological, and environmental factors.Within this framework, a role for early life stressors has been proposed. Although this association is probably not specific to Bipolar Disorders BDs, with early life stressors predisposing to trans nosological indicators for severity of psychiatric disorders, it may represent an early marker both for triggering and long-term clinical manifestations of BDs.

Insomnia likely plays a triggering role in the onset and maintenance of BDs, as it may might play a key role in BDs by potentially dysregulating the systems involved in mood and emotion regulation, including stress and inflammatory systems and circadian rhythms alterations. Early life stressors have been demonstrated to alter sleep regulation. It has been hypothesized that sleep disruption related to early life stressors might contribute to the development pathways towards BDs in adult life through the epigenetic re-reprogramming of stress and inflammatory systems.

**Objectives:**

We aimed to study their association with mood symptoms and suicidal risk in BDs with and without clinically significant insomnia symptoms. Since hopelessness is a construct strongly predicting depressive symptoms and suicidal risk in BDs and it has also been related to early life stress and to insomnia symptoms we aimed to specifically assess this symptom in our research.

**Methods:**

a sample of 162 adult participants with BD I or II were assessed during depressed phase using the Structural Clinical Interview for DSM-5 (SCID-5), the Beck Depression Inventory-II (BDI-II), the Young Mania Rating Scale (YMRS), the Early Trauma Inventory Self Report-Short Form (ETISR-SF), the Beck Hopelessness Scale (BHS), the Insomnia Severity Index (ISI) and the Scale for Suicide Ideation (SSI). Participants with or without clinically significant insomnia were compared and we carried out correlations, regression and mediation analyses.

**Results:**

Participants with insomnia showed a greater severity of depressive symptoms,of suicidal risk, of the cognitive component of hopelessness and of early life stressors. Insomnia symptoms mediated the association among early life stress and depressive symptoms (Z = 2.72, p = 0.0006), the cognitive component of hopelessness (Z = 3.02, p = 0.0001) and suicidal ideation and plans (Z = 2.07 p = 0.0006).

**Conclusions:**

fnsomnia may mediate the relationship between early life stress and clinical manifestations of BD. Assessing the evolution of insomnia symptoms could offer an approach to characterize BD and to formulate treatment strategies. In particular targeting insomnia symptoms might potentially modify the clinical features of BD in response to early life stressful events.

**Disclosure of Interest:**

None Declared

